# Supplementary material for: Somatic mosaicism in ALS and FTD identifies focal mutations associated with widespread degeneration
Source: Nat Genet. 2026 Apr 15;58(5):1019–29. doi: 10.1038/s41588-026-02570-6 (PMC13175891; doi:10.1038/s41588-026-02570-6)
Supplement: Supplementary file 1 — Supplementary Figs. 1–3 and Supplementary Note. [file 41588_2026_2570_MOESM1_ESM.pdf]

nature genetics

Supplementary information https://doi.org/10.1038/s41588-026-02570-6

Somatic mosaicism in ALS and FTD identifies focal mutations associated with widespread degeneration

In the format provided by the authors and unedited

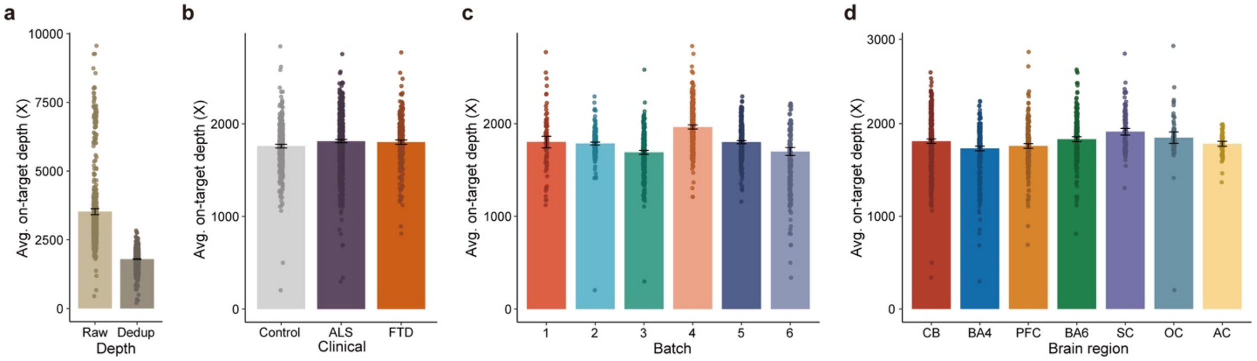

Supplementary Fig. 1: Depth distribution of MIP panel sequencing data. Average sequencing depth of the data is depicted across different conditions: (a) before and after UMI deduplication (n=1,787), (b) across clinical conditions (n=516, 938, and 375, respectively; 42 samples are included in both ALS and FTD as they originate from ALS-FTD patients), (c) by sequencing batch (n=91, 307, 358, 378, 378, and 275, respectively), and (d) across brain regions (n=529, 393, 295, 290, 132, 75, and 73, respectively). All samples represent biological replicates. Bar graph, mean ± 95% CI.

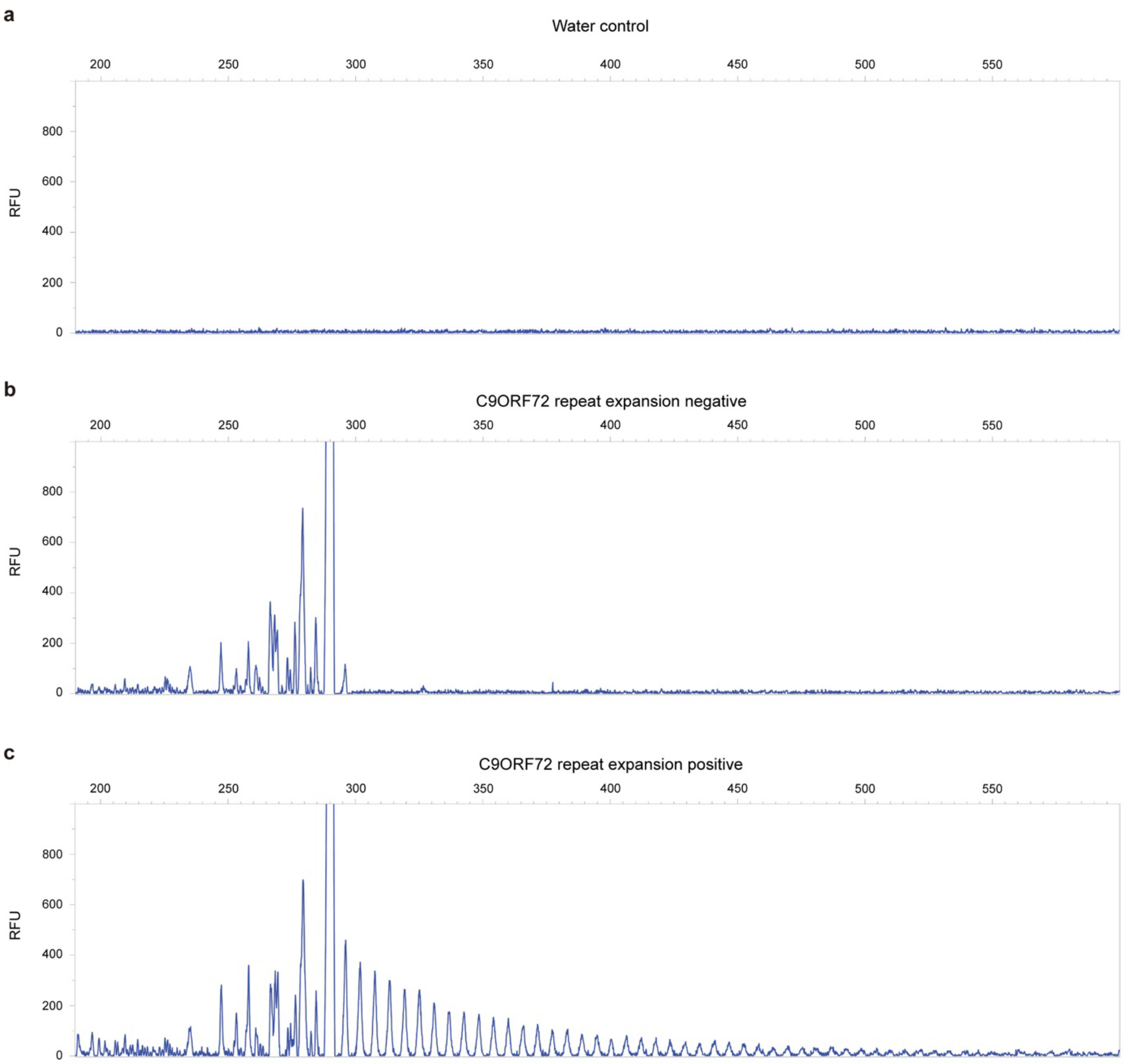

Supplementary Fig. 2: Genotyping of *C9ORF72* hexanucleotide repeat expansions for ALS and FTD cases using repeat-primed PCR assays. The presence of *C9ORF72* hexanucleotide repeat expansions in 291 ALS and 117 FTD cases was assessed by repeat-primed PCR assays. (a) The electropherogram of a water control without *C9ORF72* hexanucleotide repeat expansions. (b) A negative sample with a low number of *C9ORF72* hexanucleotide repeat expansions. (c) A positive sample with a large number of *C9ORF72* hexanucleotide repeat expansions. RFU: Relative fluorescence units. X-axis denotes the fragment size (bp).

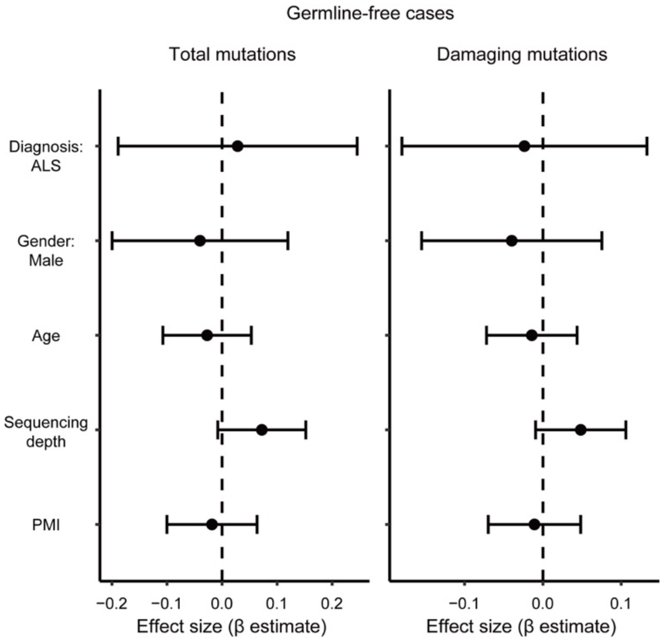

Supplementary Fig. 3: Analysis of somatic SNVs in bulk RNA-seq data of brain and spinal cord tissues of sALS cases detected by RNA-MosaicHunter. Linear regression modeling did not find a significant enrichment of somatic SNVs in sALS cases that lack pathogenic germline variants in ALS genes. PMI, post-mortem interval. Bulk RNA-seq data from 682 tissues of 143 sALS cases and 107 tissues of 23 control cases were analyzed. The significance of enrichment and 95% CI were estimated while controlling for potential confounding factors including gender, age, and sequencing and sample qualities using a linear mixed model.

Supplementary Note

MIP panel design and sequencing of postmortem tissues from diseased and control individuals

We designed a MIP panel targeting the exons and exon-intron junctions of 88 neurodegeneration/dementia-related genes, which included 34 ALS/FTD genes, 10 Alzheimer's disease genes, 28 Parkinson's disease genes, and 16 genes associated with other rare neurodegenerative disorders (Supplementary Table 2). We performed MIP panel sequencing at ~1,800X average sequencing depth after deduplication based on unique molecular identifiers (UMIs) (Fig. 1b and Supplementary Fig. 1), with a similar distribution of sequencing depth across batches, disease conditions, and tissue regions (Supplementary Fig. 1). The variance of depth, along with the batch and sample information, were considered as covariates in the variant burden test. A total of 938, 375, and 516 samples from 291 ALS, 117 FTD, and 144 neurotypical control individuals respectively were sequenced (Fig. 1a, 1c and Supplementary Table 1). Of the ALS and FTD cases, nine were diagnosed with both ALS and FTD. Therefore, 42 samples from these cases were included in both conditions, leading to a total of 1,787 unique samples.

Benchmarking of the custom pipeline for somatic variant calling

We performed spike-in experiments by mixing two human samples from the Genome in a Bottle Consortium (GIAB) at VAFs of 10%, 5%, 2.5%, 1%, and 0.5% and estimated theoretical sensitivity across sequencing depths (Extended Data Fig. 1a, b; see Methods). Double-called variants identified by Mutect2 and Pisces were excluded from the final call set due to high false positive and low validation rates (Extended Data Fig. 1c, d). High sensitivity and precision were achieved for the remaining Replow-based double-called variants (Replow-Mutect2 and Replow-Pisces) while maintaining a low false positive rate across the low VAFs compared to the somatic variants called by each caller. The MIP sequencing and our custom pipeline together allowed us to confidently identify somatic variants with a low false positive rate at VAF as low as 0.5%. The observed VAFs of somatic variants were well in line with the target VAFs at all five VAF levels.

Detection of somatic variant candidates arising from sample contamination

We identified low-level DNA contamination derived from another sample in 29 out of 1,787 samples (13 ALS, 10 FTD, and 6 control samples). Germline variants from the contaminant mimicked low-VAF somatic variants, leading to false positive calls. To address this, we implemented a module to identify low-level contamination and filter out candidates originating from the contaminant. By comparing the somatic candidate set of a given sample with the germline call set of other individuals, sample contamination was determined if the sample had ≥ 40 low-VAF somatic candidates that were also observed as germline variants in another specific individual. In such cases, the germline variants of the matched individual were considered as potential sources of false positive calls, and all matching somatic candidates of the contaminated sample were filtered out. After this filtration, six of the 29 samples still harbored a total of seven somatic variant candidates. To ensure these were not artifacts of contamination, the remaining seven candidates from the contaminated samples were reexamined to verify the absence of corresponding variants within the contaminant sources, confirming these as independent candidates.

Hypodiploid nuclei indicate apoptotic cells

Previous flow cytometric studies have established that reduced DNA content, leading to a hypodiploid cell population, is a hallmark of late-stage apoptosis, primarily due to leakage of endonuclease-cleaved DNA fragments. These fragmented cells form a distinct hypodiploid population, often referred to as the "sub-G1" peak.

Burden analysis of somatic variants using linear mixed model

For both MIP and RNA sequencing data, linear mixed-effect regression models (linear mixed models) were used to evaluate the relationships between somatic variant burden and clinical conditions, while accounting for other covariates that may affect the burden. An individual-level analysis framework was used, in which candidate somatic variants were aggregated across all tissue samples from each individual. Individual-wise variant lists were constructed by merging all candidate variants from the same donor, with overlapping variants across multiple tissue samples counted only once to avoid overestimation. A linear mixed-effect regression model was then applied to evaluate associations between somatic variant burden and clinical conditions, while adjusting for potential covariates. Clinical conditions and covariates of interest—including sex, postmortem interval (PMI), average sequencing depth, and the number of samples per donor—were modeled as fixed effects. Sequencing batch (batch 1 to 6) was modeled as a random effect to account for variation introduced by sample clustering within the same batch.

The somatic variant burden per individual was modeled as: $y_{ij}=\mu+\alpha_i+\beta_i+\gamma_i+\delta_i+n_i+U_i+\varepsilon_i$, where y_i is the somatic variant burden of donor i (normalized per megabase), μ is the average variant burden in the normal condition, α_i is the fixed effect of PMI, β_i is the fixed effect of disease status (ALS or FTD) compared to normal controls, γ_i is the fixed effect of sex, δ_i is the fixed effect of average sequencing depth across all samples from donor i, and n_i is the fixed effect accounting for the number of samples per donor. $U_{ij}\sim N(0, \sigma_r^2)$ represents the random effect of sequencing batch, and $\varepsilon_i\sim N(0, \sigma^2)$ is the residual error term. A covariate with a p-value < 0.05 was considered to be significant, based on a t-test using the Satterthwaite approximation of degrees of freedom. To test the burden of somatic variants in different genomic regions, a linear mixed model was fitted to the corresponding variant counts of specific type (e.g. exonic). To test the burden of somatic variants in different brain regions, samples were first divided by the sequenced region and then a linear mixed model was fitted for each region group. All models were fitted using R (v4.1.0) with the lme4 (v1.1.30) and the lmerTest (v3.1.3) R packages.
