# Supplementary material for: Somatic mosaicism in ALS and FTD identifies focal mutations associated with widespread degeneration
Source: Nat Genet. 2026 Apr 15;58(5):1019–29. doi: 10.1038/s41588-026-02570-6 (PMC13175891; doi:10.1038/s41588-026-02570-6)
Supplement: Supplementary file 2 — Reporting Summary [file 41588_2026_2570_MOESM2_ESM.pdf]

nature portfolio

Corresponding author(s): Clotilde Lagier-Tourenne, Eunjung Alice Lee and Christopher A. Walsh

Last updated by author(s): Feb 18, 2026

Reporting Summary

Nature Portfolio wishes to improve the reproducibility of the work that we publish. This form provides structure for consistency and transparency in reporting. For further information on Nature Portfolio policies, see our Editorial Policies and the Editorial Policy Checklist.

Statistics

For all statistical analyses, confirm that the following items are present in the figure legend, table legend, main text, or Methods section.

n/a	Confirmed	
☐	☒	The exact sample size (n) for each experimental group/condition, given as a discrete number and unit of measurement
☒	☐	A statement on whether measurements were taken from distinct samples or whether the same sample was measured repeatedly
☐	☒	The statistical test(s) used AND whether they are one- or two-sided<br>*Only common tests should be described solely by name; describe more complex techniques in the Methods section.*
☐	☒	A description of all covariates tested
☐	☒	A description of any assumptions or corrections, such as tests of normality and adjustment for multiple comparisons
☐	☒	A full description of the statistical parameters including central tendency (e.g. means) or other basic estimates (e.g. regression coefficient) AND variation (e.g. standard deviation) or associated estimates of uncertainty (e.g. confidence intervals)
☐	☒	For null hypothesis testing, the test statistic (e.g. F, t, r) with confidence intervals, effect sizes, degrees of freedom and P value noted<br>*Give P values as exact values whenever suitable.*
☒	☐	For Bayesian analysis, information on the choice of priors and Markov chain Monte Carlo settings
☒	☐	For hierarchical and complex designs, identification of the appropriate level for tests and full reporting of outcomes
☐	☒	Estimates of effect sizes (e.g. Cohen's d, Pearson's r), indicating how they were calculated

Our web collection on statistics for biologists contains articles on many of the points above.

Software and code

Policy information about availability of computer code

Data collection	No collection software was used.
Data analysis	(1) MIP targeted gene panel sequencing analysis: Sequencing data was processed to generate analysis-ready BAM using BWA-mem (0.7.15), samtools (1.3.1), and GATK (3.7) as described in Methods section. Cutadapt (2.4), BAMClipper (1.0.1), and UMI-tools (1.0.0) were also used to perform preprocessing steps including adapter trimming, read masking, and UMI deduplication. GATK (3.7), RePlow (1.1.0), Mutect2 (4.1.5), and Pisces (5.2.11) were used to identify SNV and indel candidates from MIP sequencing data. ANNOVAR (2017Jul17) was used to deliver computational information of multiple databases for variant pathogenicity prediction. All data visualization and statistical tests including linear mixed-effect regression modelling were performed using R software (4.0.1). The implemented codes for data preprocessing, statistical test, and visualization will be available before publication.<br>(2) Bulk RNA-seq analysis: STAR (v2.5.0a) was used for read alignment. Picard (1.138) and GATK (3.6) were used for duplicates removal, SplitNCigarReads, indel realignment, and base quality recalibration. RNA-MosaicHunter was used to call somatic SNVs, and its source code and default configuration file are available at https://gitlab.aleelab.net/august/rna-mosaichunter.git.<br>(3) Targeted long-read sequencing analysis: Raw HiFi reads were aligned to the human reference genome (GRCh38) using minimap2 (v2.28), and sorting and indexing were performed using samtools (v1.3.1). The Tandem Repeat Genotyping Tool (TRGT, v1.1.1) was used to determine the C9ORF72 repeat counts for each sample (chr9:27573528-27573546, (GGGGCC)n). A waterfall plot was generated using TRVZ to visualize repeat counts with mosaicism.<br>(4) Repeat-primed PCR assay: The RP-PCR products were separated by the SeqStudio Genetic Analyzer (Thermo Fisher) with the GeneScan™ 600 LIZ™ Dye Size Standard (Thermo Fisher). Results of fragment sizes were analyzed by Peak Scanner™ Software v1.0 (Thermo Fisher).

For manuscripts utilizing custom algorithms or software that are central to the research but not yet described in published literature, software must be made available to editors and reviewers. We strongly encourage code deposition in a community repository (e.g. GitHub). See the Nature Portfolio guidelines for submitting code & software for further information.

Data

Policy information about availability of data

All manuscripts must include a data availability statement. This statement should provide the following information, where applicable:

- Accession codes, unique identifiers, or web links for publicly available datasets
- A description of any restrictions on data availability
- For clinical datasets or third party data, please ensure that the statement adheres to our policy

The bulk RNA-seq data generated by the NYGC ALS Consortium are available through controlled access via the Target ALS Data Portal (https://dataengine.targetals.org/). Access requires acceptance of the Target ALS Data Use Agreement and submission of a data access request through the portal application process. Additional details are provided in the Target ALS Data Portal User Manual. The MIP-based targeted sequencing data and long-read sequencing data generated in this study have been deposited in dbGaP under accession number phs003530, with access governed by human subject privacy regulations. Germline and somatic variants identified and validated in this study are listed in the supplementary tables.

Research involving human participants, their data, or biological material

Policy information about studies with human participants or human data. See also policy information about sex, gender (identity/presentation), and sexual orientation and race, ethnicity and racism.

Reporting on sex and gender	The cases involved in this study were selected regardless of the sex and gender. The sex and gender were unintentionally balanced. We did not perform specific analyses to compare differences between genders.
Reporting on race, ethnicity, or other socially relevant groupings	Race and ethnicity information was not available for all cases involved in this study. Cases were selected regardless of the race and ethnicity. No related analyses were performed.
Population characteristics	Sporadic ALS and FTD cases were selected based on available clinical records provided by brain banks. ALS and FTD cases without clear recording of family histories were also selected if the age of death was above 45 years old. Normal cases did not have dementia or other neurological diseases based on available clinical records.
Recruitment	The study did not involve live human participants. Case selection is described above.
Ethics oversight	Tissue collection and distribution for research and publication was conducted according to protocols approved by the the Massachusetts Alzheimer's Disease Research Center, Oxford Brain Bank, Target ALS Foundation affiliated brain banks , and NIH NeuroBioBank affiliated brain banks. Research on these deidentified specimens and data was performed at Boston Children's Hospital with approval from the Committee on Clinical Investigation.

Note that full information on the approval of the study protocol must also be provided in the manuscript.

Field-specific reporting

Please select the one below that is the best fit for your research. If you are not sure, read the appropriate sections before making your selection.

☒ Life sciences ☐ Behavioural & social sciences ☐ Ecological, evolutionary & environmental sciences

For a reference copy of the document with all sections, see nature.com/documents/nr-reporting-summary-flat.pdf

Life sciences study design

All studies must disclose on these points even when the disclosure is negative.

Sample size	No sample-size calculation was performed. We tried to collect as many sporadic ALS, FTD and control cases as possible. Our results indicate that the sample size is enough to make statistically significant conclusions.
Data exclusions	No data was excluded.
Replication	Germline mutations identified in this study were shared by multiple tissue samples from the same individuals, which validates the germline mutations. Forty one somatic mutations identified in the MIP panel sequencing data were further validated by amplicon sequencing. Thirty four of these somatic mutations were also validated by ddPCR. The DYNC1H1 and LMNA somatic mutations identified in bulk RNA-seq data were also validated by amplicon sequencing.
Randomization	Cases were classified into ALS, FTD and control groups based on their clinical records and diagnoses. Clinical conditions and covariates of interest (e.g. age, gender, sequencing depth) were modeled as fixed effects and the batch and individual (donor) information were modeled as random effects, considering the uncertainty caused by sample clusters from the same origin (donor or batch).
Blinding	No blinding was performed. Blinding was not relevant because samples were postmortem tissues obtained from established brain banks, and downstream sequencing, variant calling, and validation were performed using automated pipelines with predefined criteria independent of disease status.

Reporting for specific materials, systems and methods

We require information from authors about some types of materials, experimental systems and methods used in many studies. Here, indicate whether each material, system or method listed is relevant to your study. If you are not sure if a list item applies to your research, read the appropriate section before selecting a response.

Materials & experimental systems

n/a	Involved in the study
☐	☒ Antibodies
☐	☐ Eukaryotic cell lines
☐	☐ Palaeontology and archaeology
☐	☐ Animals and other organisms
☐	☐ Clinical data
☐	☐ Dual use research of concern
☐	☐ Plants

Methods

n/a	Involved in the study
☐	☐ ChIP-seq
☐	☒ Flow cytometry
☐	☐ MRI-based neuroimaging

Antibodies

Antibodies used	NeuN (Millipore, MAB377, clone A60, 1:1,500) pTDP-43 (CosmoBio, CAC-TIP-PTD-P03, polyclonal, 1:10,000)
Validation	The NeuN antibody has been validated for use in FC, IC, IF, IH, IH(P), IP and WB. Its species reactivity has been validated for Av, Ch, Ft, H, M, Po, R, and Sal. This antibody has been cited in more than one thousand publications. All the information is available on the manufacturer's website. The pTDP-43 antibody has been validated for use in WB, ELISA and IHC for detection of human TDP-43 phosphorylated on serine 409.

Eukaryotic cell lines

Policy information about cell lines and Sex and Gender in Research

Cell line source(s)	n/a
Authentication	n/a
Mycoplasma contamination	n/a
Commonly misidentified lines (See ICLAC register)	n/a

Palaeontology and Archaeology

Specimen provenance	n/a
Specimen deposition	n/a
Dating methods	n/a

☐ Tick this box to confirm that the raw and calibrated dates are available in the paper or in Supplementary Information.

Ethics oversight	n/a

Note that full information on the approval of the study protocol must also be provided in the manuscript.

Animals and other research organisms

Policy information about studies involving animals; ARRIVE guidelines recommended for reporting animal research, and Sex and Gender in Research

Laboratory animals	n/a
Wild animals	n/a
Reporting on sex	n/a

April 2023

Field-collected samples | n/a

Ethics oversight | n/a

Note that full information on the approval of the study protocol must also be provided in the manuscript.

Clinical data

Policy information about clinical studies

All manuscripts should comply with the ICMJE guidelines for publication of clinical research and a completed CONSORT checklist must be included with all submissions.

Clinical trial registration	n/a
Study protocol	n/a
Data collection	n/a
Outcomes	n/a

Dual use research of concern

Policy information about dual use research of concern

Hazards

Could the accidental, deliberate or reckless misuse of agents or technologies generated in the work, or the application of information presented in the manuscript, pose a threat to:

No	Yes	
☒	☐	Public health
☒	☐	National security
☒	☐	Crops and/or livestock
☒	☐	Ecosystems
☒	☐	Any other significant area

Experiments of concern

Does the work involve any of these experiments of concern:

No	Yes	
☒	☐	Demonstrate how to render a vaccine ineffective
☒	☐	Confer resistance to therapeutically useful antibiotics or antiviral agents
☒	☐	Enhance the virulence of a pathogen or render a nonpathogen virulent
☒	☐	Increase transmissibility of a pathogen
☒	☐	Alter the host range of a pathogen
☒	☐	Enable evasion of diagnostic/detection modalities
☒	☐	Enable the weaponization of a biological agent or toxin
☒	☐	Any other potentially harmful combination of experiments and agents

Plants

Seed stocks	n/a
Novel plant genotypes	n/a
Authentication	n/a

ChIP-seq

Data deposition

☐ Confirm that both raw and final processed data have been deposited in a public database such as GEO.

☐ Confirm that you have deposited or provided access to graph files (e.g. BED files) for the called peaks.

Data access links<br>*May remain private before publication.*	n/a
Files in database submission	n/a
Genome browser session<br>(e.g. UCSC)	n/a

Methodology

Replicates	n/a
Sequencing depth	n/a
Antibodies	n/a
Peak calling parameters	n/a
Data quality	n/a
Software	n/a

Flow Cytometry

Plots

Confirm that:

☒ The axis labels state the marker and fluorochrome used (e.g. CD4-FITC).

☒ The axis scales are clearly visible. Include numbers along axes only for bottom left plot of group (a 'group' is an analysis of identical markers).

☒ All plots are contour plots with outliers or pseudocolor plots.

☒ A numerical value for number of cells or percentage (with statistics) is provided.

Methodology

Sample preparation	Postmortem brain tissues were homogenized and lysed followed by sucrose gradient based nuclei isolation. Nuclei were stained with NeuN antibody and DAPI before FACS sorting.
Instrument	BD FACSAria II
Software	BD FACSDiva
Cell population abundance	The purify of NeuN+ neurons was not examined in this study. Our previous studies using the same gating strategy showed >96% purity of neurons.
Gating strategy	The gating strategy is shown in Extended Data Fig. 6.

☒ Tick this box to confirm that a figure exemplifying the gating strategy is provided in the Supplementary Information.

Magnetic resonance imaging

Experimental design

Design type	n/a
Design specifications	n/a
Behavioral performance measures	n/a

Acquisition

Imaging type(s)	n/a
Field strength	n/a
Sequence & imaging parameters	n/a
Area of acquisition	n/a

Diffusion MRI ☐ Used ☒ Not used

Preprocessing

Preprocessing software	n/a
Normalization	n/a
Normalization template	n/a
Noise and artifact removal	n/a
Volume censoring	n/a

Statistical modeling & inference

Model type and settings	n/a
Effect(s) tested	n/a

Specify type of analysis: ☐ Whole brain ☐ ROI-based ☐ Both

Statistic type for inference (See Eklund et al. 2016)	n/a
Correction	n/a

Models & analysis

n/a	Involved in the study
☒	☐ Functional and/or effective connectivity
☒	☐ Graph analysis
☒	☐ Multivariate modeling or predictive analysis
